# Development of Injectable Fucoidan and Biological Macromolecules Hybrid Hydrogels for Intra-Articular Delivery of Platelet-Rich Plasma

**DOI:** 10.3390/md17040236

**Published:** 2019-04-19

**Authors:** Hsien-Tsung Lu, Wan-Ting Chang, Min-Lang Tsai, Chien-Ho Chen, Wei-Yu Chen, Fwu-Long Mi

**Affiliations:** 1 School of Biomedical Engineering, College of Biomedical Engineering, Taipei Medical University, Taipei 11031, Taiwan; 2 Department of Orthopedics, Taipei Medical University Hospital, Taipei 11031, Taiwan; 3 Graduate Institute of Medical Sciences, College of Medicine, Taipei Medical University, Taipei 11031, Taiwan; wendy1104000@yahoo.com.tw; 4 Department of Food Science, National Taiwan Ocean University, Keelung 20224, Taiwan; tml@mail.ntou.edu.tw; 5 School of Medical Laboratory Science and Biotechnology, College of Medical Science and Technology, Taipei Medical University, Taipei 11031, Taiwan; chenchho@tmu.edu.tw; 6 Department of Pathology, School of Medicine, College of Medicine, Taipei Medical University, Taipei 11031, Taiwan; 1047@tmu.edu.tw; 7 Department of Pathology, Wan Fang Hospital, Taipei 11696, Taiwan; 8 Department of Biochemistry and Molecular Cell Biology, School of medicine, College of Medicine, Taipei Medical University, Taipei 11031, Taiwan; 9 Graduate Institute of Nanomedicine and Medical Engineering, College of Biomedical Engineering, Taipei Medical University, Taipei 11031, Taiwan

**Keywords:** fucoidan, genipin, hydrogels, drug delivery, growth factors, platelet-rich plasma

## Abstract

Platelet-rich plasma (PRP) is rich in growth factors and has commonly been utilized in the repair and regeneration of damaged articular cartilage. However, the major drawbacks of direct PRP injection are unstable biological fixation and fast or burst release of growth factors. Fucoidan is a heparinoid compound that can bind growth factors to control their release rate. Furthermore, fucoidan can reduce arthritis through suppressing inflammatory responses and thus it has been reported to prevent the progression of osteoarthritis, promote bone regeneration and accelerate healing of cartilage injury. Injectable hydrogels can be used to deliver cells and growth factors for an alternative, less invasive treatment of cartilage defects. In this study, hyaluronic acid (HA) and fucoidan (FD) was blended with gelatin (GLT) and the GLT/HA/FD hybrid was further cross-linked with genipin (GP) to prepare injectable GP-GLT/HA/FD hydrogels. The gelation rate was affected by the GP, GLT, HA and FD concentrations, as well as the pH values. The addition of HA and FD to GLT networks improved the mechanical strength of the hydrogels and facilitated the sustained release of PRP growth factors. The GP-GLT/HA/FD hydrogel showed adequate injectability, shape-persistent property and strong adhesive ability, and was more resistant to enzymatic degradation. The PRP-loaded GP-GLT/HA/FD hydrogel promoted cartilage regeneration in rabbits, which may lead to an advanced PRP therapy for enhancing cartilage repair.

## 1. Introduction

Articular cartilage injury caused by trauma and osteoarthritis often leads to disability; however, the avascular nature of cartilage limits its self-healing ability. Therefore, articular cartilage repair and regeneration continue to be challenged. Platelet-rich plasma (PRP) has gained much attention as a potential material for repairing damaged cartilage. PRP is an autologous enriched source of various growth factors which has been utilized for clinical cartilage repair, resulting in improvements in recovering the function of articular cartilage. However, the common use of PRP injection for articular cartilage repair often has limited efficacy due to its rapid clearance from the injection site. Recent studies for advanced therapy to repair cartilage defects have demonstrated that the controlled release of PRP from various hydrogels and scaffolds enhanced chondrogenic differentiation and promoted cartilage defect repair [1,2,3,4].

Fucoidan is a sulfated polysaccharide extracted from marine algae which exerted a wide variety of pharmacological activities including antitumor [5,6], angiogenesis [7,8], anti-inflammatory [9,10,11,12], antioxidant [13], anticoagulant and antithrombotic [14,15,16], antiviral and antibacterial [17,18], and osteogenic differentiation activities [19]. Fucoidan is a safe, non-toxic and biocompatible material [20], which has been used to develop nanomedicine for drug delivery and magnetic resonance imaging [21,22,23,24,25,26], and to prepare scaffolds for bone [27,28,29] and cartilage [30,31] tissue engineerings. Fucoidan improves meniscal injury [32], reduces the severity of arthritis through the suppression of Th1-mediated immune reactions [33], and lowers serum TNF-α, IL-1β and MMP-1 [34]. In particular, fucoidan is an active selectin blocker which can reduce postischemic inflammatory cascades and prevent inflammatory damage [35]. These studies revealed that fucoidan has the potential to suppress inflammatory arthritis and reduce cartilage damage.

Fucoidan is a heparin-like molecule that modulates the effect of a variety of growth factors on cell proliferation to promote osteogenic differentiation [36], angiogensis and revascularization [37], and improves the bioactivity of mesenchymal stem cells [38]. Several studies have reported that fucoidan can interact with growth factors to control their release and activity through binding to growth factors and regulation of the signaling pathways. Various drug delivery systems based on fucoidan were developed to locally concentrate and slowly release transforming growth factor (TGF-β), bone morphogenic protein 2 (BMP2), vascular endothelial growth factor (VEGF), basic fibroblast growth factor (bFGF) and stromal cell-derived factor-1α (SDF-1α), including fucoidan-based scaffolds [39,40,41], electrospun fibers [42], bioprostheses [43] and nanoparticles [38,44], to promote endothelial cell migration and VEGF-mediated angiogenesis [40,42], fibroblasts migration [41] and neurite extension [44], which improved the early step of cardiac differentiation from human embryonic stem cells [39], increased the antithrombotic and re-endothelialization potential of bioprostheses [43], and stimulated the mobilization of mesenchymal stem cells [38]. On the basis of the fact that PRP is rich in high concentrations of various growth factors, fucoidan is a potential biological macromolecule for intra-articular delivery of PRP.

Articular cartilage is a hyaline tissue composed of chondrocytes and extracellular matrix (ECM) components such as collagen type II and glycosaminoglycans (GAGs). Hyaluronic acid (HA) is an important GAG in ECM of articular cartilage, which plays a vital role in enhancing the viscoelastic properties of cartilage tissue to reduce the friction at joints by the release of compressive stress. Gelatin (GLT) is obtained by partial hydrolysis of collagen, which is cheap, biocompatible, fully degradable, and absorbable and non-immunogenic, and thus is suitable for a wide range of tissue engineering applications. Genipin (GP) is a gardenia fruit extract with a natural ability to cross-link chitosan, collagen and GLT. It has markedly lower cytotoxicity (5000- to 10,000-fold) than the commonly used glutaraldehyde. Our previous study reported that the nucleophilic attack of primary amine groups on genipin resulted in the formation of cross-linked chitosan or GLT networks [45,46,47,48,49]. Reyes-Ortega reported the development of bioactive bilayered dressing containing GP-crosslinked HA/GLT hybrid hydrogels for epidermal tissue regeneration [50]. The cross-linking of the HA/GLT hybrids with GP resulted in the formation of a semi-interpenetrating network (SIPN) in which HA chains were randomly distributed in the crosslinked GLT matrix. The HA chains weren’t cross-linked but were stabilized by hydrogen bonds, electrostatic interactions, hydrophobic forces, etc. [50]. However, to the best of our knowledge, the use of GP for the preparation of injectable GLT/HA/fucoidan (FD) based hydrogels has still not been reported.

The aim of this study is, therefore, to develop GP-crosslinked GLT/HA/FD hydrogels with injectability, enzyme degradation resistance, controlled release properties, and cartilage adhesiveness for intra-articular delivery of PRP, and thus which can improve the efficiency of PRP therapy by controlling growth factor release and offer an alternative and promising approach for advanced cartilage regenerative therapy.

## 2. Results and Discussion

### 2.1. Depolymerization of Fucoidan

Low molecular weight fucoidan (LMWF) has been reported to have antioxidant and neuroprotective properties [51], antihyperglycemic, antihyperlipidemic, and hepatoprotective activities [52], and can improve cardiac function [53]. It is worth noting that LMWF is able to stabilize established atherosclerotic lesions because it has great antioxidant activity. LMWF reduces lipid peroxidation and foaming macrophage accumulation, and ameliorates the inflammatory response through down-regulation of IL-6 and up-regulation of IL-10 levels, and by returning p-JNK and cyclin D1 to normal levels [54,55,56]. Other studies reported that LMWF downregulated microparticle release and pro-inflammatory properties of activated human polymorphonuclear neutrophils [57], suppressed the inflammatory response in RAW 264.7 macrophages by inhibiting MAPK and oxidative stress [58], and reduced arthritis through suppressing the Th1 immune response [33]. On the basis of the above-mentioned advantages, LMWF was prepared and used to combine with GLT and HA for the development of injectable hydrogels.

**Figure 1:**
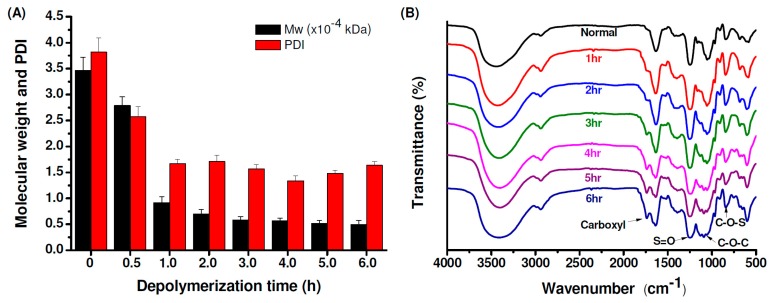
(**A**) Weight-average molecular weights (Mw) and polydispersity index (PDI) of the original and depolymerized fucoidan, (**B**) Fourier Transform-Infrared (FTIR) spectra of the original and depolymerized fucoidan.

Figure 1A shows the molecular weights and polydispersity index (PDI) of original and depolymerized fucoidan. The glycosidic bonds in fucoidan are hydrolyzed by H_2_O_2_ and then the weight average molecular weight (Mw) decreased from 34685 Da (PDI = 3.82) to 4952 Da (PDI = 1.64). Figure 1B shows the Fourier Transform-Infrared (FTIR) spectra of original and depolymerized fucoidan. The original fucoidan shows characteristic peaks at 1030 cm^−1^, 1255 cm^−1^ and 842 cm^−1^, which are assigned to the absorption of the C−O−C asymmetric stretch (glycosidic linkage), and S=O and C−O−S stretches of sulfate ester. The spectrum of the fucoidan depolymerized at a different time shows an additional peak at 1729 cm^−1^, which is assigned to the C=O stretch of carbonyl or carboxyl groups generated after depolymerization, and the peak intensity increases with the decrease of the molecular weight of the depolymerized fucoidan.

**Figure 2:**
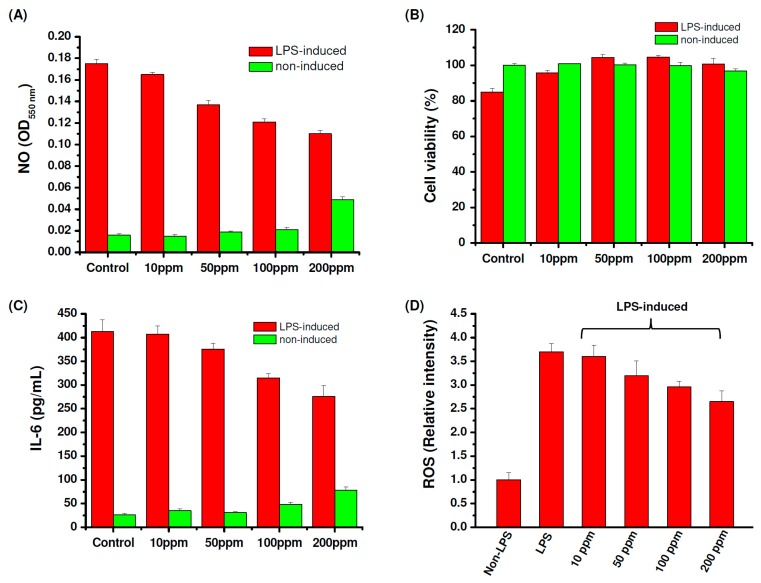
Anti-inflammatory activity of depolymerized fucoidan against lipopolysaccharides (LPS) (1 μg/mL) induced cytotoxicity in RAW 264.7 macrophages: (**A**) Nitrogen oxide (NO) production, (**B**) cell viability, (**C**) IL-6 production, (**D**) reactive oxygen species (ROS) production.

After three hours of depolymerization, the molecular weight of fucoidan showed no obvious change; thus the 3 h depolymerized fucoidan (Mw = 6984 kDa and PDI = 1.71) was selected as LMWF for the following studies. To examine whether the depolymerized fucoidan has a suitable protective effect against osteoarthritis through enhancing the inflammatory activation of macrophages, the fucoidan samples were incubated with lipopolysaccharides (LPS)-stimulated RAW 264.7 cells. Interleukin-6 (IL-6) is a cytokine closely related to arthritis which has the effect of promoting inflammation. The level of nitric oxide (NO) production in the activated macrophages stimulated by LPS is used as an index of inflammation. Fucoidan is known to downregulate inflammatory cytokines in LPS-activated macrophages and suppress NO production [12,59]. As shown in Figure 2A, the depolymerized fucoidan effectively inhibits LPS-induced NO production in a dose-dependent manner. However, when the concentration of the depolymerized fucoidan increased to 200 ppm, NO production in normal macrophages (without stimulation by LPS) clearly increased, indicating that the immune modulation effect of the depolymerized fucoidan can take place at a higher concentration. Figure 2B shows that co-culture of the LPS-stimulated RAW 264.7 macrophages with the depolymerized fucoidan increases the cell viability, revealing the protective effect of the depolymerized fucoidan against LPS-induced cytotoxicity. Moreover, the expression of the inflammatory cytokineIL-6 by LPS-stimulated macrophages was reduced by the depolymerized fucoidan, see Figure 2C. The results suggest that the depolymerized fucoidan is effective in reducing the LPS-induced inflammatory response in the activated macrophages, leading to the reduction of the expression of pro-inflammatory cytokine and mediator, IL-6 and NO.

NO produced by the immunologic isoform of iNOS triggers a rapid release of reactive oxygen species (ROS), which can cause oxidative damage in osteoarthritic cartilage. Increased ROS production is known to induce DNA damage, apoptosis, and cartilage degradation of chondrocytes, which may cause the deterioration of osteoarthritis [60]. Therefore, we further investigated the ROS scavenging activity of the depolymerized fucoidan. ROS production by the activated macrophages stimulated by LPS was determined by ROS-sensitive fluorescence dye (DCFDA). The activated macrophages treated with the depolymerized fucoidan effectively inhibited the ROS production induced by LPS, see Figure 2D. The depolymerized fucoidan seems to be a promising material to inhibit the ROS produced by inflammatory macrophages. On the basis of the previous results, we select the depolymerized fucoidan (Mw = 6984 kDa and PDI = 1.71) to be combined with GLT and HA for the following preparation of PRP-loaded injectable hydrogels.

### 2.2. Cross-Linking Reaction and Gel Formation

**Figure 3:**
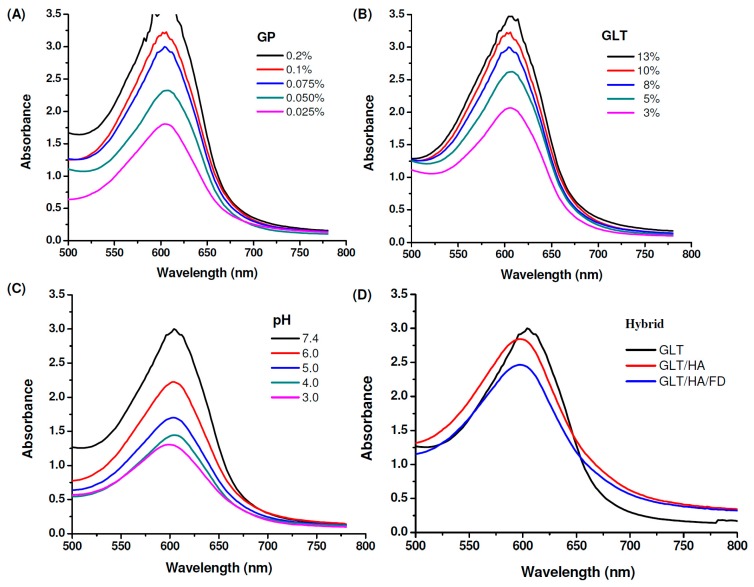
Absorption spectra of genipin (GP)-crosslinked gelatin (GLT), GLT/hyaluronic acid (HA) and GLT/HA/ fucoidan (FD) at 37 °C: (**A**) 8% GLT cross-linked with different concentrations of GP at pH 7.4, (**B**) different concentrations of GLT cross-linked with 0.075% GP at pH 7.4, (**C**) 8% GLT cross-linked with 0.075% GP at different pH values, (**D**) 8% GLT cross-linked with 0.075% GP at pH 7.4 in the presence of 1% HA and FD.

It has been reported that a GP-amine monomer is formed via a nucleophilic attack by an amino group containing compounds such as GLT on the olefinic carbon at C-3 on deoxyloganin aglycone of GP [47,49]. This reaction is followed by the opening of the GP ring, allowing a secondary amine attack on the resulting aldehyde. GLT reacts with GP to form a blue pigment, which can be used as an index of the cross-linking progress. The ultraviolet–visible (UV-Vis) spectrum of GP in water displayed an absorption peak at 240 nm. The GP-crosslinked compounds showed two new absorption peaks at 280 nm (π − π*) and 600 nm (n − π*) due to the formation of GP cross-linked heterocyclic amines [47,49,61]. The GP and GLT concentration-dependent effects appeared as indicated by increasing the intensity of the absorbance at 600 nm with the increase of the concentrations of GP and GLT, respectively, see Figure 3A,B. Higher concentrations of GLT or GP allowed greater interactions between GLT and GP, resulting in a greater number of chemical crosslinks being formed. The GP-crosslinking rate of GLT is strongly influenced by pH (pH 3, 4, 5, 6 and 7.4), see Figure 3C. In general, the intensity of the absorbance at 600 nm increased upon increasing the pH because of the reduction in the degree of protonation. Furthermore, the hydroxide ion generated in phosphate buffered saline (PBS) caused GP to form aldehyde groups [62], allowing GP to cross-link GLT at a higher rate. GLT/HA hybrids were prepared by blending GLT with HA, followed by cross-linking the hybrids with GP, resulting in the formation of a GP-crosslinked GLT/HA semi-interpenetrating polymer network (semi-IPN) [50].

Figure 3D shows that the intensity of the absorbance at 600 nm decreases in the presence of 1 wt% HA and 1 wt% depolymerized fucoidan (FD), revealing that the cross-linking reactions of the GLT/HA and GLT/HA/FD hybrids occur at a slower rate than that of GLT only. HA and FD are acidic polysaccharides which can release H^+^, and thus the pH levels of the GLT/HA and GLT/HA/FD hybrids are lower than their GLT counterpart. We have shown that the GP-involved cross-linking reaction rate increased with increasing pH, see Figure 3C. Thus, the reason for the slower color changing rates of the GLT/HA and GLT/HA/FD hybrids can be attributed to the decrease of pH by HA and FD. Furthermore, the HA and FD macromolecular chains interpenetrating within the GLT network structure may reduce the cross-linking reactivity between GP and GLT.

**Figure 4:**
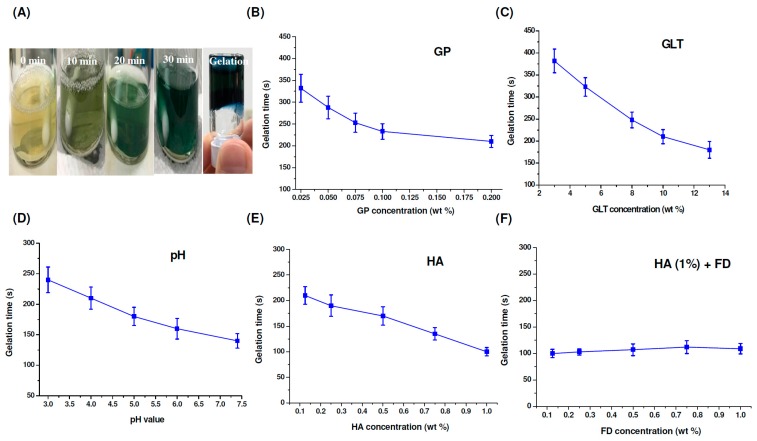
(**A**) Color changes and inverted tube test of the GP-crosslinked GLT samples, (**B**–**E**) gelation times of GP-crosslinked GLT, GLT/HA and GLT/HA/FD at 37 °C: (**B**) 8% GLT cross-linked with different concentrations of GP at pH 7.4, (**C**) different concentrations of GLT cross-linked with 0.075% GP at pH 7.4, (**D**) 8% GLT cross-linked with 0.075% GP at different pH values, (**E**) 8% GLT cross-linked with 0.075% GP at pH 7.4 in the presence of different concentrations of HA, (**F**) 8% GLT cross-linked with 0.075% GP at pH 7.4 in the presence of 1% HA and different concentrations of FD.

As shown in Figure 4A, after cross-linking GLT (8%) with GP (0.075%), the color changed from yellow to green to blue. The gelation time of the GP-crosslinked samples can be determined by using the inverted tube test at 37 °C. The results showed that the GP, GLT and HA concentrations, as well as the pH, played vital roles in gel formation. The gelation time decreased from 323 min to 210 min and from 382 min to 180 min with the increase of GP concentration from 0.025% to 0.2% and GLT concentration from 3% to 13%, see Figure 4B,C. Changing the pH value from 3.0 to 7.4 (the physiological condition) caused a faster gelation rate, see Figure 4D, and thus the gelation time decreased from 240 min to 150 min. The effects of GP and GLT concentrations and pH value on the gelation time correlated with the results of spectral changes during the gel formation process, see Figure 3A–C. However, the gelation time decreased from 210 min to 100 min with the increase of HA concentration from 0.125% to 1.0%, see Figure 4E. The tendency is opposite to that of spectral change, see Figure 3D. Luo et al. reported that 1-Ethyl-3-(3-dimethylaminopropyl)-carbodiimide (EDC)/ N-hydroxysuccinimide (NHS)-crosslinked GLT/HA hybrid injectable hydrogel has higher elasticity compared to the EDC/NHS-crosslinked GLT hydrogel [63]. Accordingly, the faster gelation rates of the GP-crosslinked GLT/HA hybrid at higher HA contents could be attributed to the superior elastic property of HA. Figure 4F shows the gelation time of the GP-crosslinked GLT/HA/FD hybrids. The gelation rates were only slightly affected by FD at different concentrations when the concentration of HA was kept at 1 wt%. Fucoidan solution has a low apparent viscosity and poor viscoelastic properties [21], and thus the polymer doesn’t have thickening, gelling or film-forming properties. On the basis of the results, we concluded that the gelation times of the GP-crosslinked GLT/HA/FD hybrids were significantly affected by HA but only slightly affected by FD.

### 2.3. Injectability, Stability and Adhesive Property

**Figure 5:**
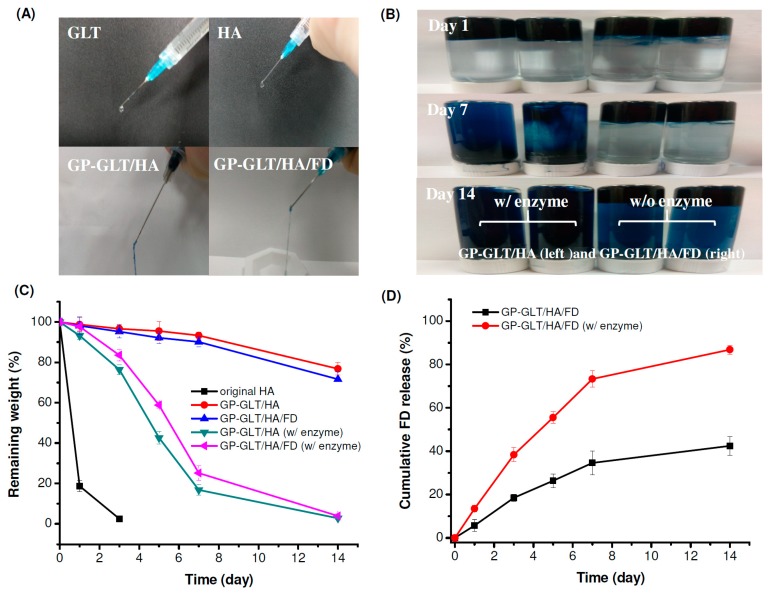
Injectability, stability, degradability and FD release property of GP-crosslinked GLT/HA and GLT/HA/FD hybrid hydrogels (GP-GLT/HA and GP-GLT/HA/FD) prepared at 37 °C: (**A**) injectability of the hydrogels through a 23-gauge needle, (**B**) stability of the hydrogels after injection into phosphate buffered saline (PBS) with (w/) and without (w/o) enzyme, (**C**) weight loss of the hydrogels in PBS with (w/) and without enzyme, (**D**) FD release from the hydrogels in PBS with (w/) and without enzymes.

The original GLT and HA solutions were squeezed through the syringe with a 23 G needle to produce liquid-like droplets. In contrast, the GP-crosslinked GLT/HA and GLT/HA/FD hybrids that were squeezed through the syringe with the same size needle were gel-like fluids, see Figure 5A, revealing the formation of injectable hydrogels. Next, the hydrogels were directly injected into PBS to examine whether the hydrogels are strong enough to stay firm in physiological fluids such as joint fluid after injection. Figure 5B shows that the hydrogels injected through the 23 G needle into PBS solution retained their shape, indicating that the hydrogels were shape-persistent materials. It is known that hydrogen bonding formed in hydrogels can be broken by H_2_O molecules; therefore, weakening the adhesion force of the hydrogels. The hydrogels prepared in this work were adhesive and attached to the bottom side when injected into PBS. The gels were still attached to the bottom of the bottle after 14 days, even after turning the bottles upside down, see Figure 5B. The injectability, and shape-persistent and adhesive properties suggest that the injectable hydrogels can be used for intra-articular delivery of cells, growth factors and PRP for cartilage repair.

As mentioned above, the GP-crosslinked GLT/HA (GP-GLT/HA) and GP-crosslinked GLT/HA/FD (GP-GLT/HA/FD) hydrogels were adhesive and very stable in PBS. We further examined the weight loss of the hydrogels in PBS with (w/) and without (w/o) enzymes, see Figure 5C. The original HA without cross-linking or further treatments was quickly degraded by hyaluronidase. The GP-GLT/HA and GP-GLT/HA/FD hydrogels exhibited slower degradation rates. The lower weight loss of the hybrid hydrogels was attributed to the higher heterogeneity of the GP-GLT/HA and GP-GLT/HA/FD semi-interpenetrating polymer networks compared to the original HA [64], which reduced the susceptibility of the incorporated HA to proteolytic degradation.

### 2.4. Rheological Property, Compressive Strength and Enzymatic Degradation

**Figure 6:**
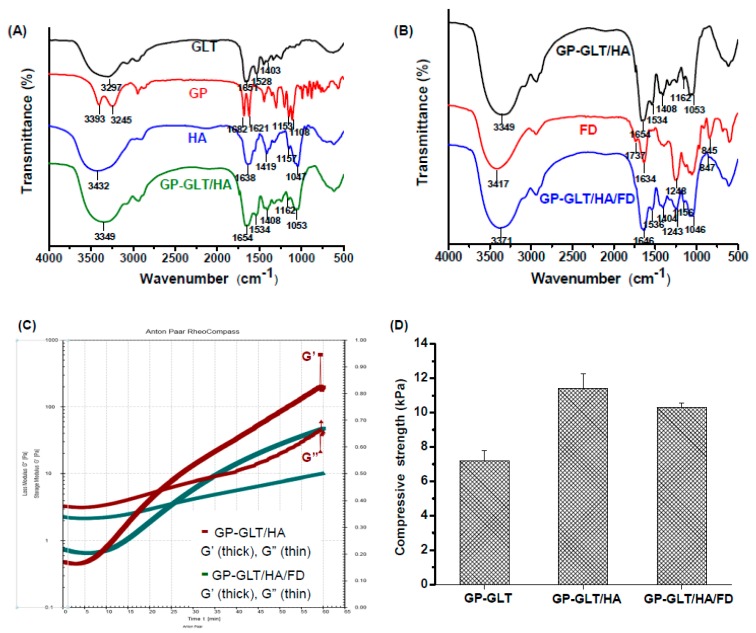
Chemical and physical properties of GP-crosslinked GLT/HA and GLT/HA/FD hybrid hydrogels (GP-GLT/HA and GP-GLT/HA/FD): (**A**) FTIR spectra of GP, GLT, HA and GP-GLT/HA, (**B**) FTIR spectra of FD, GP-GLT/HA and GP-GLT/HA/FD, (**C**) rheological behavior, (**D**) compressive strength.

Figure 6A shows the FTIR spectra of GP, GLT, HA and the GP-crosslinked GLT/HA hydrogel (GP-GLT/HA). The spectrum of HA shows the absorption peaks at 1419 cm^−1^ and 1638 cm^−1^, which are assigned to the symmetric and asymmetric stretching of carboxylate groups. The peaks that appeared in the range of 1000–1200 cm^−1^ (1047 cm^−1^ and 1157 cm^−1^) are assigned to the C−O−C and C−O stretching of the HA saccharide ring. GLT clearly shows two characteristic peaks at 1651 cm^−1^ and 1528 cm^−1^, corresponding to amide I (C=O stretching) and amide II (N−H deformation). GP shows two characteristic peaks at 1682 and 1621 cm^−1^ which are assigned to the C=O stretching of ester groups and the C=C stretching of the dihydropyran ring. Furthermore, the C−O−C and C−O stretching bands at 1108 cm^−1^ and 1153 cm^−1^ are attributed to the ether and hydroxyl groups in dihydropyran ring. GP-GLT/HA hydrogel shows the characteristic peaks of the HA saccharide ring (1053 cm^−1^ and 1162 cm^−1^), and the characteristic peaks of GLT amide I and II bands (1654 cm^−1^ and 1534 cm^−1^) which are overlapped with the absorption peaks of HA carboxyl groups. The peaks located around 1408 cm^−1^ and 3349 cm^-1^ were attributed to the overlap of the absorption peaks of HA carboxylate groups and GLT alkyl groups (1403 cm^−1^), and the overlap of the absorption peaks assigned to the amino (N−H stretching) and hydroxyl groups (O−H stretching) of HA (3432 cm^−1^) and GLT (3297 cm^−1^). The characteristic peaks of GP were not obvious in GP-GLT/HA hydrogel because the GP concentration (0.075%) was much lower than those of GLT, HA and FD. Compared with the GP-GLT/HA hydrogel, the GP-GLT/HA/FD hydrogel shows the characteristic absorption bands of fucoidan at 1243 cm^−1^ (asymmetrical stretching of S=O), and 847 cm^−1^ (sulfate ester), see Figure 6B, confirming the incorporation of fucoidan in the hybrid hydrogel.

The gel formation process of the GP-GLT/HA and GP-GLT/HA/FD hydrogels was monitored by using rheology testing, see Figure 6C. The storage modulus (G′) of the GP-GLT/HA hybrid increased faster than its GP-GLT/HA/FD counterpart. This was caused by the elastic property of HA, which increased the gel strength by reinforcement of the GP-crosslinked GLT network structure [65]. The storage modulus (G′) of the samples was, in the end, higher than the storage modulus (G″). The intersection of G′ and G″ indicates the point that the elastic solid-like behavior began to exceed the viscous liquid-like behavior. By monitoring the intersection of G′ and G″, the best time for mixing cells or growth factors can be accurately predicted. This provided us with useful information for mixing the GLT/HA and GLT/HA/FD hybrids with PRP before gelation. The compression strength of the GP-GLT/HA hydrogel was 11.4 kPa, which was higher than that of the GP-GLT hydrogel, see Figure 6D. The enhanced compression strength can be directly linked to the high elastic property of HA, which formed semi-IPNs after cross-linking. Hydrogels used in cartilage are superior if they have an elastic property as well as suitable viscosity and stiffness, which are critical to their ability to resist compressive loads [65]. The GP-GLT/HA/FD hydrogel possesses a slightly lower compression strength compared with its GP-GLT/HA hydrogel counterpart. The adequate compressive strength of the hybrid hydrogels makes the hydrogels suitable for cartilage tissue applications.

### 2.5. Cytotoxicity and Growth Factor Release

**Figure 7:**
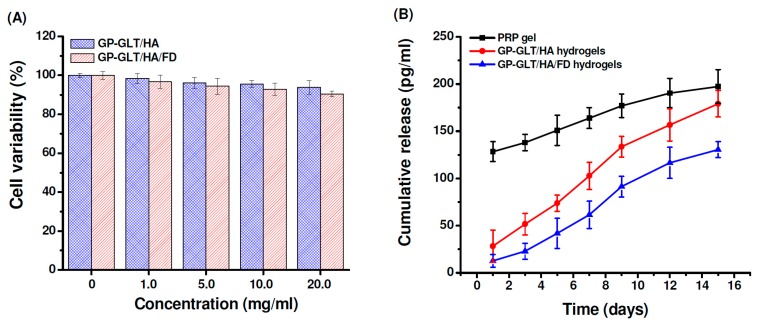
(**A**) Cytotoxicity of GP-crosslinked GLT/HA and GLT/HA/FD hybrid hydrogels, (**B**) PDGF release behavior of GP-crosslinked GLT/HA and GLT/HA/FD hybrid hydrogels.

To evaluate the cytotoxicity, the chondrocytes were treated with the GP-GLT/HA and GP-GLT/HA/FD hydrogel extracts at different concentrations. Cell viability was not evidently influenced by the extract at concentrations below 20 mg/mL, indicating no significant toxicity of the hydrogels (Figure 7A). This is attributed to the reason that GP, GLT, HA and FD are well known to be nontoxic and biocompatible. PRP gel traditionally prepared through thrombin activation usually releases incorporated growth factors with burst effects, resulting in the decrease of its therapeutic efficacy. Platelet-derived growth factor (PDGF) is one of the important growth factors in PRP for cartilage defect repair [1,2,3,4]. As shown in Figure 7B, PDGF was released slowly from the GP-GLT/HA hydrogels, in contrast to the burst PDGF release from the traditional PRP gel. PDGF release from the GP-GLT/HA/FD hydrogel was even slower than its GP-GLT/HA counterpart (P < 0.05) because FD is a heparinoid compound that can bind heparin-binding growth factors to control their release rate. PDGF was known to bind to heparin-like molecules mainly via electrostatic interaction [66]. Liu et al. reported that the proliferation of chondrocyte was enhanced by a photo-crosslinkable PRP-loaded hydrogel which could release growth factors in a more sustained manner [2,4]. The cartilage regeneration efficiencies of the PRP-loaded GP-GLT/HA/FD hydrogels were then investigated in a rabbit cartilage defect model.

### 2.6. In Vivo Histological Evaluation

**Figure 8:**
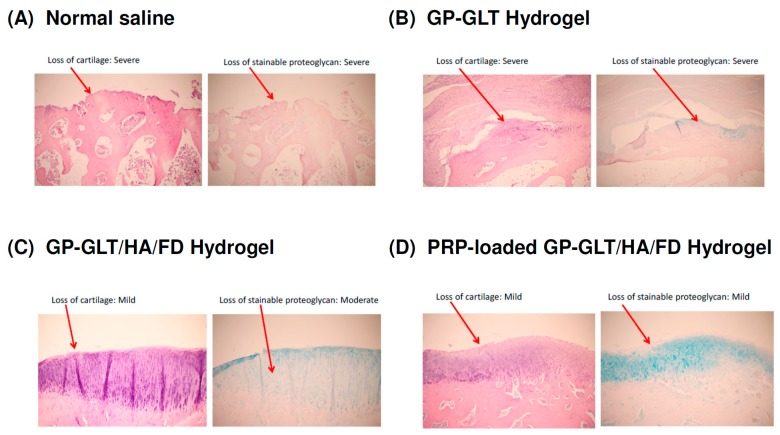
Histological examinations of cartilage at the femoral condyles after treatments by intra-articular injection of: (**A**) normal saline (control), (**B**) GP-GLT hydrogel, (**C**) GP-GLT/HA/FD hydrogel, (**D**) PRP-loaded GP-GLT/HA/FD hydrogel.

The macroscopic examination of the control group (intra-articular injection of normal saline) showed that the femoral of the articular cartilage was rough and dull (data not shown). Histological analysis suggested that the intra-articular injection of the GP-GLT/HA/FD hydrogel or PRP-loaded GP-GLT/HA/FD hydrogel attenuated the losses of chondrocytes at the femoral condyles. The animals treated with either the GP-GLT/HA/FD hydrogel or PRP-loaded GP-GLT/HA/FD hydrogel showed decreased lesion formation and reduced losses of proteoglycans in the superficial layer, see Figure 8C,D, compared with the control and the GP-GLT hydrogel treated groups, see Figure 8A,B). Overall, the results suggest that the treatment with intra-articular injection of GP-GLT/HA/FD hydrogel or PRP-loaded GP-GLT/HA/FD hydrogel may represent a desirable therapy on osteoarthritis (OA).

HA can bind chondrocytes via CD44, which regulates chondrocyte proliferation, ECM remodeling and cartilage regeneration and enhances cell differentiation into the chondrogenic phenotype. PRP-loaded hydrogel glue with the controlled release of growth factors promoted cartilage regeneration [2,4]. FD has anti-inflammatory activity [33,34,35,55,56,57,58,59] and can bind the growth factors in PRP to control their release rates [37,38,39,40,41,42,43,44,66]. Our animal study proved that GP-GLT/HA/FD hydrogel and PRP-loaded GP-GLT/HA/FD hydrogel treatments reduced cartilage degradation at the femoral condyles, and effectively reduced the loss of the superficial layer, cartilage ulceration, the production of osteophytes, the creation of fissures, and the disorganization of cartilage, compared with the control (intra-articular injection of normal saline) and GP-GLT hydrogel treated groups. The use of PRP in tissue regeneration has been developed for clinical applications. Intra-articular injection of GP-GLT/HA/FD hydrogel or PRP-loaded GP-GLT/HA/FD hydrogel reduced the degradation of cartilage on the femoral condyles, and effectively reduced the loss of the superficial layer, compared with the control and GP-GLT-hydrogel-treated groups. The losses of chondrocytes and proteoglycan of the groups treated by intra-articular injection of normal saline and GP-GLT hydrogel were higher than those of the GP-GLT/HA/FD and PRP-loaded GP-GLT/HA/FD hydrogel groups.

## 3. Materials and Methods

### 3.1. Materials

HA was purchased from SeedChem Company PTY., LTD, Victoria, Australia. Fucoidan from *Laminaria japonica* was purchased from NOVA Pharma & Liposome Biotech (Kaohsiung, Taiwan). GP was purchased from Challenge Bioproducts Co., Ltd. (Touliu, Taiwan). GLT (225 g Bloom, type B, suitable for cell culture), 3-(4,5-dimethylthiazol-2-yl)-2,5-diphenyl tetrazolium bromide (MTT), 2′,7′-Dichlorodihydrofluorescein diacetate (DCFDA), gelatinase and hyaluronidase were purchased from Sigma−Aldrich, Louis, MO, USA.

### 3.2. Preparation of LMWF

Fucoidan (0.2 g) was dissolved in 200 mL deionized water. Then, H_2_O_2_ was added to the fucoidan solution at a final concentration of 0.2 M. The fucoidan/H_2_O_2_ mixed solution was continuously stirred under magnetic stirring at 70 °C for 1–6 h, followed by terminating the reaction using 0.1 M sodium carbonate. The samples after depolymerization were dialyzed against deionized water using a dialysis tubing (MWCO 1000 Da). The molecular weight of depolymerized, low molecular weight fucoidan (LMWF) was determined by gel permeation chromatography (GPC) with a TSKgel G3000PWXL column (Tokyo, Japan). Detector: refractive index detector (RI); Mobile phase: Na_2_SO_4_ aqueous solution (0.2 M); Flow rate: 0.5 mL/min; Column temperature 40 °C. Dextran standards of different weights from PSS Polymer Standards Service GmbH (Mainz, Germany) were used to construct standard curves.

### 3.3. Characterization of Anti-Inflammatory and ROS Scavenging Effects

#### 3.3.1. Colorimetric Nitric Oxide Assay

RAW 264.7 cells (1 × 10^4^ cells/mL) were stimulated with LPS (1 μg/mL) for 24 h in the presence or absence of the depolymerized fucoidan (Mw = 6984 kDa). Nitric oxide produced by RAW 264.7 macophage was measured by the Griess reagent. Briefly, 50 μL of Griess solution was added to the same volume of cell culture medium and then was further incubated for 10 min. The absorbance at 540 nm was measured by a EnSpire2300 multimode plate reader (Perkin Elmer, Waltham, MA, USA) to determine the nitrite concentration from a sodium nitrite standard curve.

#### 3.3.2. Enzyme-Linked Immunosorbent Assay (ELISA) for IL-6

After 24 h of stimulation of RAW 264.7 cells with LPS in the presence or absence of the depolymerized fucoidan, the cell culture supernatants were collected and the concentrations of IL-6 were measured by ELISA kits (R&D Systems Inc., Minneapolis, MN, USA) following the manufacturer’s instructions.

#### 3.3.3. Analysis of ROS Generation

After 24 h of stimulation of RAW 264.7 cells with LPS in the presence or absence of the depolymerized fucoidan, the cell culture supernatants were collected and then 10 μM DCFDA was added to the supernatants. The generated ROS concentration was determined by measuring the fluorescence intensity of DCF (DCFDA was oxidized by ROS to form DCF) by a Perkin Elmer EnSpire2300 multimode plate reader (Ex 485 nm, Em 520 nm).

### 3.4. Preparation of Injectable Hydrogels

GLT solutions were prepared by dissolving different amounts of GLT in 100 mL distilled water to obtain the desired GLT concentrations (3, 5, 8, 10 and 13 wt%). GLT/HA and GLT/HA/FD blends were prepared by mixing HA and FD with GLT in distilled water at various weight ratios (0.125, 0.25, 0.5, 0.75, 1.0 wt%) to obtain GLT/HA and GLT/HA/FD hybrid hydrogels. Different amounts of GP were then added to the GLT, GLT/HA and GLT/HA/FD solutions to reach the final GP concentrations (0.025, 0.05, 0.075, 0.1, 0.2 wt%). The pH was adjusted to 3.0, 4.0, 5.0, 6.0 and 7.4.

### 3.5. UV-Vis Absorption Spectra

The cross-linking reaction was analyzed by dissolved GP in GLT, and GLT/HA and GLT/HA/FD hybrid solutions at the above-mentioned concentrations and pH, followed by stirring at room temperature. UV-Vis absorption spectra were recorded by Perkin Elmer EnSpire2300 multimode plate reader.

### 3.6. Fourier Transform Infrared Spectroscopy (FTIR)

To investigate the chemical structures of GP-crosslinked GLT, GLT/HA and GLT/HA/FD hydrogels (GP-GLT, GP-GLT/HA and GP-GLT/HA/FD), the test samples were dried and ground to a powder. The FT-IR spectra of HA, GLT, GP-GLT, GP-GLT/HA and GP-GLT/HA/FD were recorded using a Perkin Elmer, RXI FTIR System in the region between 400–4000 cm^−1^.

### 3.7. Rheological Characterization

Rheological properties were measured using a parallel-plate rheometer (AR2000ex, TA Instruments, New Castle, DE, USA). A time sweep at 1 Hz of the GP-GLT/HA and GP-GLT/HA/FD solutions were determined by placing the test samples within the plates and continuously recording the storage and loss modulus values (G′ and G″) at 37 °C.

### 3.8. Gelation Time Determination

The gelation time was determined using an inverted tube test at 37 °C. Briefly, 2 mL of the GP-containing GP-GLT/HA and GP-GLT/HA/FD solutions were placed in a sample bottle (7 mL), and then, the bottles were incubated in a water bath at 37 °C. The gelation times were determined by inclining the bottle every 30 s until the gel did not flow.

### 3.9. Compressive Mechanical Properties

The GP-GLT/HA and GP-GLT/HA/FD solutions added with an adequate amount of GP were placed in 24-well plates and left to cross-link for the formation of hydrogels. The compression tests of the hydrogel samples were performed on a Texture Profile Analyzer (Model TA-XT2; Stable Micro Systems Ltd, Surrey, UK with a cylinder load of 1 g moving at 0.1 mm/s to plot the pressure (Pa) as a function of strain. The hydrogel sample was compressed to 80% of the initial height.

### 3.10. Cytotoxicity

Human chondrocyte primary cells were purchased from Cell Application, Inc. (San Diego, CA, USA). The cytotoxicity of the hydrogels was evaluated according to the ISO 10993-5 Standard. Briefly, GP-GLT/HA and GP-GLT/HA/FD hydrogels were incubated at 37 °C in PBS buffer (pH 7.4, 10 mL) for 24 h. The collected solutions were mixed with culture medium (1:1) and then were cultured with chondrocyte (5 × 10^3^ cells per well) for 48 h. An MTT assay was conducted to evaluate cell viability by measuring the absorbance of the supernatant solution at 540 nm using a EnSpire 2300 multimode plate reader (Perkin Elmer, Waltham, MA, USA).

### 3.11. In Vitro Biodegradation Study

The degradation study was performed by firstly weighing the hydrogels after gel formation. Subsequently, the hydrogels were incubated at 37 °C in PBS buffer with gentle agitation in an orbital shaker and the mass loss was determined at a predetermined time period by removing the hydrogels from the medium, blotting the hydrogels with a filter paper, and finally weighing the hydrogels. The PBS buffer containing 10 U/mL hyaluronidase and 0.5 U/mL gelatinase was used for enzymatic degradation studies. After that, the medium was replaced with fresh PBS or enzyme solution. The degradation ratio was expressed as the percentage of weight loss.

### 3.12. PRP-Loading and Release

The lyophilized PRP powder was prepared using RegenKit THT Autologous Platelet-Rich Plasma (A-PRP) (RegenKit® THT, Stryker, Kalamazoo, Michigan, USA). The PRP-loaded hydrogels were prepared by adding PRP in GP-GLT/HA and GP-GLT/HA/FD before gelation. The PRP-loaded hydrogels were placed in a 24-well plate containing 500 μL of release medium (PBS). The release medium was collected at a predetermined time interval and then the content of PDGF was determined using ELISA kit (R&D Ltd, Minneapolis, MN, USA).

### 3.13. Animal Model and Design

This study was conducted with the permission of the Experimental Animal Review Committee of Taipei Medical University. In one knee of each of the New Zealand white rabbits, the anterior cruciate ligament was transected (ACLT) to create the experimental osteoarthritis (OA) model [67]. The rabbits were kept within the guidelines of IACUC without extra calcium provided. Three rabbits with ACLT that underwent intra-articular normal saline injection were used as a control group. The treatment group used nine rabbits from three groups that were injected intra-articularly using either GP-GLT hydrogel, GP-GLT/HA/FD hydrogel or PRP-loaded GP-GLT/HA/FD hydrogel.

### 3.14. Specimen Collection and Histological (Microscopic) Examination

After 8 weeks of administration, the rabbits were euthanized and then specimen collection and histological (microscopic) examination were performed according to the methods reported in our previous paper [67]. Hematoxyline and eosin stain (H&E stain) and alcian blue stain of the samples were used for detection of glycosaminoglycans (GAG).

### 3.15. Statistical Analysis

All data are presented as the arithmetic mean ± standard deviation (SD). Statistical analysis was performed using a Student’s *t*-test. The data were considered to be significantly different at P < 0.05.

## 4. Conclusions

In summary, we have developed an injectable hydrogel based on blending HA and fucoidan (FD) with GLT followed by cross-linking the GLT/HA/FD hybrid with GP. The concentrations of GP, GLT, HA and FD affected the gelation rates of the hydrogels. The addition of HA and FD enabled high strength, high stability, and strong adhesive ability. Furthermore, the GP-GLT/HA/FD hydrogels were more resistant to enzymatic degradation and had a more sustained release of growth factors. Because of the growth factor-binding characteristic of FD, the PRP-loaded hydrogel can overcome the drawbacks of direct PRP injection and is helpful in cartilage defect regeneration. The PRP-loaded hydrogel promoted cartilage regeneration in a rabbit cartilage defect model, which assured us the material had potential for the treatment of cartilage injury.
